# Replication initiatives will not salvage the trustworthiness of psychology

**DOI:** 10.1186/s40359-016-0134-3

**Published:** 2016-05-31

**Authors:** James C. Coyne

**Affiliations:** Department of Health Psychology, University Medical Center, University of Groningen, P.O. Box 196, 9700 AD Groningen, The Netherlands

**Keywords:** Reproducibility, p-hacking, Randomized controlled trials, Publication bias

## Abstract

Replication initiatives in psychology continue to gather considerable attention from far outside the field, as well as controversy from within. Some accomplishments of these initiatives are noted, but this article focuses on why they do not provide a general solution for what ails psychology. There are inherent limitations to mass replications ever being conducted in many areas of psychology, both in terms of their practicality and their prospects for improving the science. Unnecessary compromises were built into the ground rules for design and publication of the Open Science Collaboration: Psychology that undermine its effectiveness. Some ground rules could actually be flipped into guidance for how *not* to conduct replications. Greater adherence to best publication practices, transparency in the design and publishing of research, strengthening of independent post-publication peer review and firmer enforcement of rules about data sharing and declarations of conflict of interest would make many replications unnecessary. Yet, it has been difficult to move beyond simple endorsement of these measures to consistent implementation. Given the strong institutional support for questionable publication practices, progress will depend on effective individual and collective use of social media to expose lapses and demand reform. Some recent incidents highlight the necessity of this.

*Bad publication practices keep good scientists unnecessarily busy, as in replicability projects.- Björn Brembs* [1]

In revising this paper, I strengthened its focus on explaining why we cannot look to replication initiatives as the primary means of salvaging the trustworthiness of psychology. I benefited from two excellent reviews that encouraged me to pursue this theme. I interpreted the reviewers’ comments as a prompt to specify the vantage point from which I view replication initiatives: that of a stakeholder in improving the trustworthiness of the psychological literature who is outside of personality and social psychology, the areas of psychology to which replication initiatives are most relevant. I also had the benefit of an unfolding controversy generated by a critical commentary [2] and a response [3] from some of the authors of the Open Science Collaboration’s (OSC) Replication Project: Psychology article in *Science* [4] and, in particular, the debate that this exchange prompted in social media.

## Point of view

I am a clinical health psychologist and mental health researcher. While I continue to publish peer-reviewed articles, I have increasingly turned my attention to (1) blogging and activism to improve the trustworthiness of biomedicine and science, particularly psychology; (2) generating an appropriate skepticism about the literature and its portrayal in the media; and (3) fostering a citizen-scientist orientation in consumers, arming them with the tools to decide for themselves the credibility of the advice being offered to them, and further tools to access and evaluate information that is beyond their expertise, but nonetheless required for their personal decision-making.

In my early career as a faculty member at University of California, Berkeley, my colleagues in personality and social psychology tried to persuade me that they were the real scientists of the field. Even if I was a clinical *research* psychologist, they argued, I was too concerned with practical issues, whereas they were concerned with the theory and methodology on which I would depend. They actually convinced me that the relatively smaller grants that they were receiving were more significant than the usually larger National Institute of Health grants, because their grants came from the more prestigious National *Science* Foundation.

As an outside stakeholder, I now view personality and social psychology as a source of frustration. Andrew Gelman [5] captures one half of my annoyance:

“All sorts of ridiculous studies on topics such as political moderation and shades of gray, or power pose, or fat arms and political attitudes, or ovulation and vote preference, or ovulation and clothing, or beauty and sex ratios, or elderly-related words and walking speed, or subliminal smiley faces and attitudes toward immigration, or ESP in college students, or baseball players with K in their names being more likely to strike out, or brain scans and political orientation, or the Bible Code, are getting published in top journals and getting lots of publicity. Indeed, respected organizations such as the Association for Psychological Science and the British Psychological Society have promoted what I (and many others) would consider junk science.”

I would go further. Articles consist of small groupings of small studies assembled by an unknown sampling of the studies run in a lab. Dependent measures are typically of unestablished internal or external validity, other than they have been previously used. Most measures are obtained by self-report, making the data vulnerable to demand characteristics and extraneous influences. The experimental manipulations are of untested external validity, but the widest generalizability is claimed. There’s an overrepresentation of results just barely achieving statistical significance, which is of dubious importance anyway, except for satisfying the confirmation bias required for publication [6]. From my vantage point, these problems are compounded by the publicity machines of professional organizations and journals screaming “Listen up consumers, here are scientific results that you must accommodate in your life.” The press releases are meant to be digested in the moment and forgotten. But checking claims made a few years ago destroys any credibility that might be accorded to current claims. For instance, consider a 2011 press release from the Association for Psychological Science [6], “Life is one big priming experiment”:

“Scientists have shown again and again that they can very subtly cue people’s unconscious minds to think and act certain ways. These cues might be concepts—like cold or fast or elderly—or they might be goals like professional success; either way, these signals shape our behavior, often without any awareness that we are being manipulated.

This is humbling, especially when you think about what it means for our everyday beliefs and actions. The priming experiments take place in laboratories, using deliberately contrived signals, but in fact our world is full of cues that act on our minds all the time, for better or for worse. Indeed, many of our actions are reactions to random stimuli outside our consciousness, meaning that the lives we lead are much more automated than we like to acknowledge.”

Many of us familiar with such priming research would attach little weight to whether findings of such studies can be directly replicated, although it would be not be surprising if they were not. Indeed, one of the shortcomings of the RP:P initiative is that it had no provision for screening out candidates for replication for which a consensus could be reached that the research hypotheses were improbable and not warranting the effort and resources required for a replication to establish this. Inclusion of such studies led to a waste of resources replicating bad research and a challenge to the credibility of the generalizability of claims based on what was replicated.

Personality and social psychologists have no monopoly on *questionable research practices,* but the prevalence and salience of such studies in the literature can be attributed in part to institutional agenda and *questionable publication practices*, to which they passively and actively give assent as authors, reviewers, and editors. Minimally, they give far too much praise and are too silent at the wrong times in the face of a recurring pattern.

The second half of my frustration is the rampant undeclared conflict of interests when personality and social psychologists are aided by professional organizations and journals in making money and accruing other benefits from publishing and promoting findings of questionable quality and substantive significance. In the typical case, a manuscript that is a dubious collection of flawed and underpowered studies slips through peer review and is published, with many of its initial flaws intact. Its early release on the Internet is coordinated with press releases from professional organizations, journals, and the authors themselves and their press agents. Op ed pieces and derivative articles in the media, particularly business- and corporate -oriented media, echo and even “churnal” the carefully crafted publicity. The pinnacle of success for personality and social psychologists seems to be a TED Talk, which provides credibility to claims even exceeding publication in a peer-reviewed paper. The timing of such talks often fuels suspicion that they too are part of the larger publicity effort promoting not only career advancement, but financially rewarding products. Soon after the publicizing of early release of articles comes dependably the launch of commercial products such as corporate speaking gigs, self-help books, and workshops. In such cases, I don’t recall the original peer-reviewed articles ever been accompanied by declarations of conflicts of interest to alert readers to the obvious possible risk of bias.

If this sounds like a jaded viewpoint, note that the APS press release “Life is one big priming experiment” had, embedded in it, a click link for purchasing a book by the press release’s author. A more recent example is the self-help book, *Rethinking Positive Thinking* [7], promoted in a featured book signing at the APS annual convention, as well as web links in an advertisement for the British Psychological Society Division of Health Psychology Annual Meeting. Googling the title will reveal a remarkable network of coordinated publicity fitting the pattern described above. The website dedicated to promoting the book (http://www.woopmylife.org/) lists over a dozen articles in major media outlets, along with gushing endorsements from other personality and social psychologists who, if one Googles them, have their own self-help products to promote. Elsewhere [8] I have provided a critique of the quality of the research that is cited in this book and its relevance to self-improvement and health promotion.

As I cultivate skepticism in consumers and provide them with tools to decide for themselves about the trustworthiness of what they read, I find that such coordinated efforts seemingly marshaling the endorsements of professional organizations in the name of science are disempowering and disorienting. The initiatives are calculated to shame consumers for not doing the best that they could in terms of physical and mental health and material well-being. The solution being offered is that they buy the dubious products that are being offered with the branding of being more sciencey than similar goods, as evidenced by publications in peer reviewed journals and being singled out for praise by scientific organizations and those same journals.

I regularly critique the studies associated with these initiatives in my blog posts, usually having no problem finding obvious scientific flaws and inevitable exaggeration. Too often, I cannot resist the quixotic urge to challenge particularly outrageous claims. In doing so in conventional channels, like letters to the editor and occasional calls for retraction, I renew my awareness of how resistant the personality and social psychology literature is to post publication peer review and self-correction. I return to that topic later.

## Two cheers for replication initiatives

Doubts about the trustworthiness the psychological literature cannot be euphemized away as only the “false-positives-reduction side*”* of the “evidentiary value movement” helping psychology converge on truth over time [9] or outright dispensed with the denialism of Gilbert et al.’s claims that “the reproducibility of psychological science is quite high.” [2]. On the other hand, the RP:P authors’ response [3] to Gilbert et al.’s criticism is hardly satisfying: “Using the Reproducibility Project: Psychology data, both optimistic and pessimistic conclusions about reproducibility are possible, and neither are yet warranted.”

The RP:P article in *Science* [4] is nonetheless remarkable for having selected 100 studies from three prestigious psychological journals and negotiated the original authors’ involvement in an attempt to replicate a large number of findings. Outside stakeholders will note the narrow range of subfields of psychology providing the studies and, of necessity, the narrow range of methodologies – survey, simple computer-administrative tasks, and internet studies using college students or internet samples. The simplistic literal interpretation is that most reported findings in this sample proved exaggerated or false, but we cannot know how widely to generalize or the source or practical significance of this finding. Outside stakeholders should resist making any quantitative generalization from the RP:P to the untrustworthiness of the psychological literature in general. But on the face of it, Gilbert’s [10] assertion that the results confirm “the replication rate in psychology is quite high—indeed, it is statistically indistinguishable from 100 %” seems exaggerated or outright false.

We need to appreciate some inherent limitations to replication initiatives, including the poor prospects or even desirability of extending them into all areas of psychology. As seen in the studies sampled for the recent RP:P initiative, without an extraordinary and unlikely shifting of resources, replication efforts are mainly feasible only in some areas of psychology and, in particular, readily administered surveys and laboratory tasks. This serious restriction and outright bias in coverage encourages the unwarranted assumption that these particular areas of psychology are distinctively prone to untrustworthiness of findings.

## We should not expect replication initiatives in many areas of psychology

The lack of replication initiatives for most areas of psychology is notable. It is not always for lack of efforts to organize them, but because of the formidable practical barriers that are encountered. Some represent problems in the appropriateness of replication initiatives which recur across psychology outside the narrow confines of the studies sampled by the RP:P project, but some reflect the practical issues and most pressing problems in the untrustworthiness of findings in particular areas.

### Infant studies

Many developmental psychology and infant study labs have struggled to accumulate their cohorts, facing the formidable barrier of enticing parents to give consent for their children’s participation and to return for repeated assessments. Of necessity, many infant studies remain inadequately powered, particularly those requiring delivery of complex manipulations to well defined samples. It is difficult enough to recruit and retain subjects for fresh new studies, where parent invitation and consent forms can extol the studies’ likelihood of generating new knowledge with new promises of the application of findings. It is even more of a challenge to justify studies to parents that merely test the trustworthiness of past findings.

David Peterson [11] points out that babies are more difficult to study than adults because they are more likely to fall asleep, get distracted, or put investigators’ key stimuli in their mouths. As Michael Frank [12] has aptly detailed, developmental psychology and infant studies have all the challenges of producing trustworthy findings of the rest of psychology and more:

“The average infancy study – including many I've worked on myself – has the issues we've identified in the rest of the psychology literature: low power, small samples, and undisclosed analytic flexibility. Add to this the fact that many infancy findings are never replicated, and even those that are replicated may show variable results across labs. All of these factors lead to a situation where many of our empirical findings are too weak to build theories on.

In addition, there is a second, more infancy-specific problem that I am also worried about. Small decisions in infancy research – anything from the lighting in the lab to whether the research assistant has a beard – may potentially affect data quality, because of the sensitivity of infants to minor variations in the environment. In fact, many researchers believe that there is huge intrinsic variability between developmental labs, because of unavoidable differences in methods and populations (hidden moderators). These beliefs lead to the conclusion that replication research is more difficult and less reliable with infants, but we don't have data that bear one way or the other on this question.”

Discussions on developmental psychology listserv’s and other fora have also raised questions about credit and contribution toward the professional advancement available to junior investigators participating in replication initiatives. These problems are shared with efforts to mount large-scale replication initiatives in other areas of psychology. The large numbers of authors and institutions needing to be mobilized (the RP:P article had 270 authors) make it more difficult to isolate the distinctive individual contributions needed to justify career advancement. Identifying individual contributions is always a problem for multi-author projects, but it is particularly so in research with populations requiring extensive resources to accrue sufficient numbers of subjects for adequately powered single studies. Not unreasonably, hiring and promotion decisions depend on evidence that junior faculty can independently choose research questions and methods, direct analyses, and interpret findings that advance the field.

### Clinical and health psychology interventions

The literature evaluating clinical and health psychology interventions in randomized trials has long been dominated by underpowered, low-quality studies with inadequate control groups [13, 14]. For many such interventions, there are insufficient trials to provide estimates of effect size, after the exclusion of low powered, methodologically poor studies [15, 16]. Moreover, the best predictor of the outcome of a randomized trial remains investigator allegiance [17], which accounts for 69 % of the effect size of psychotherapy outcome studies [18]. Randomized trials conducted by investigators with investments in particular treatments consistently obtain larger effect sizes, sometimes improbably large. As an example, consider the effect size obtained by promoters of problem-solving therapy for cancer patients [19]. The claimed effect size of 4.32, ten times higher than other psychological interventions, had to be excluded from meta-analyses as an extreme outlier [20, 21]. Yet, for many clinical and health psychology interventions, there are insufficient trials to provide estimates of effect size, after the exclusion of both low powered, methodologically poor studies and research conducted by those with conflicts of interest [22].

Problems in clinical trials evaluating psychological interventions were appreciated long before the development of replication initiatives. But a broad, systematic effort like the RP:P to replicate existing clinical trials of psychological interventions is not feasible and has become even less so in an era of shrinking resources for research. Of course, we should seek independent replications of individual published randomized trials wherever we can, as we always have. But that effort will be of necessity piecemeal. We need to consider the likelihood that original studies are often underpowered, and so we should discourage the common practice of relying on what are essentially pilot studies for generating estimated effect sizes, rather than simply demonstrating feasibility or acceptability [23]. We need to turn off the spigots, stop allowing small studies from entering effect sizes into the literature and withhold judgments about efficacy until the availability of larger-scale, more methodologically sophisticated studies. Any effort to simply replicate a broad array of small studies on the same scale is a distraction and even an exacerbation of the problem of consistent failures to replicate initially large effect sizes from small, methodologically poor studies conducted by investigators advocating a particular treatment.

### Observational epidemiologic studies

Large-scale observational, clinical epidemiologic studies pose special issues, particularly when the results are summoned in support of public health policies, as in attempts to reduce the adverse health consequences of obesity [24]. There have been demonstrations that the results of such observational studies can readily be manipulated to appear to favor policies preferred by the individual investigator and politicians [25]. Moreover, requirements of high impact journals that such studies specify explicitly their relevance to social and public health policies provide a strong incentive to spin results, particularly with the tight competition for the scarce space being allocated to such studies in these journals. When independent replications can be conducted in new samples, we should by all means take advantage of such opportunities. But organizing a new prospective cohort study for the specific purpose of examining a particular hypothesis with public health implications is generally not feasible in terms of time and resources.

Another example of a large and troubled area of correlational observational studies are the thousands of studies investigating depressive symptoms and other highly correlated negative affect variables [26] as a cause of death, dementia, coronary artery disease, cancer, asthma, diabetes, Parkinson’s disease, COPD, headaches, insomnia, acne, health problems after pregnancy, lower back pain, anorgasmia, premature ejaculation, impotence, hypertension, HIV viral load, poor glycemic control, constipation, diarrhea, nausea, chronic pelvic pain, incontinence, …and flatulence. Literatures are developed around specific health complaints, usually assessed by self-report. There is little effort to call attention to similar claims being made about health conditions that presumably have very different etiologies and different mechanisms by which negative emotion might conceivably influence disease status and outcome. Measures of negative affectivity are hopelessly intercorrelated among themselves, with a host of background and concurrent confounders [27, 28], which Paul Meehl has termed the “crud factor” [29]. This entire literature has been characterized as a “big mush” [30]. Do we really need attempts to replicate these studies to demonstrate that they lack value? We could do with much less of this research, particularly when it has almost always been conducted with an unknown selection of both independent and dependent variables examined in an unknown number of analyses with an unknown basis for selecting and publishing results. Under these conditions, one can even demonstrate that astrological signs have comparable associations with physical health outcomes [31].

Rather than anything resembling the RP:P model of replication initiatives, clinical epidemiological research would benefit most from publicly available cataloging of variables found in different data sets; preregistration of hypotheses and analytical strategies; fuller presentation of simple associations as well as multivariate analyses; sensitivity analyses incorporating alternative assumptions and confounds; and data sharing, particularly with an eye towards integration with other data sets so that a comparison and contrast of results can be efficiently obtained.

There will always be an inherently exploratory aspect to this line of research. Indeed, many robust results could not have been anticipated when such data were originally collected. But that literature needs to be approached with a greater recognition of the likelihood of false positives, the influence of incompletely specified and imperfectly measured confounds, and a greater resistance to acceptance of p-values as revealing anything of importance. Andrew Gelman [32] has provided a valuable elaboration of the American Statistical Association statement on p-values [33] with: “Valid p-values cannot be drawn without knowing, not just what was done with the existing data, but what the choices in data coding, exclusion, and analysis would have been, had the data been different.” A classic paper by De Groot [34] offers a relaxed view of what can be presented, but a much more restricted view of what can be taking seriously, relative to current practices: “One ‘is allowed’ to apply statistical tests in exploratory research, just as long as one realizes that they do not have evidential impact”.

## A poor model for exploring the untrustworthiness of psychology? Unnecessary and unhelpful elements built into the rules of replication initiatives

Needless compromises were built into the design and publication of the RP:P. These arbitrary procedural rules for conducting replications could be more fruitfully turned into recommendations for how *not* to conduct replications. The general theme of my objections is that collaboration initiatives, at least as they are currently organized, bureaucratize and otherwise make more complicated procedures that should be as simple as the procedures that routinely put untrustworthy science into the literature. Current rules risk ‘ghettoizing’ replications when effort should be made instead to insist on widening their acceptability, particularly in the prestigious journals that produced untrustworthy science. Furthermore, the RP:P and related initiatives inadvertently strengthen questionable publication practices which we desperately need to challenge.

### Kahneman’s adversarial collaboration

Nobel Prize winner Daniel Kahneman [35] has been influential in recommending:

“when the replication is ready – after a pilot but before data collection – the replicator sends the author a detailed description of the planned procedure, including actual programs and a video when relevant.”

And if there is any doubt in his position, he further states:

“A good-faith effort to consult with the original author should be viewed as essential to a valid replication.”

Although well-meant and intended to preempt anticipated criticism of replication initiatives, Kahneman’s [32] call for involving the authors of the original studies in replication as an adversarial collaboration is unfortunate for a number of reasons. Kahneman has provided a clear rationale for this position:

“I share the common position that replications play important role in our science – to some extent by cleaning up the scientific record, mostly by deterring soppy research. However, I believe the current norms allow replicators too much freedom to define their study is a direct replication of previous research. Authors should be guaranteed a significant role in the replications of their work.”

What could Kahneman possibly mean by “too much freedom”? Ultimately neither the original authors nor those who undertake replications have the final word on whether a study can be deemed a direct replication of previous research. That should be left to post publication peer review. Replication should be as freely undertaken as original research, and so there is no reason to slap this constraint on it. Would Kahneman extend this principle to any effort to critically examine empirically existing research findings or theoretical claims? Furthermore, if we insist on authors of original research being involved in any replications, it takes pressure off them to provide sufficiently clear and transparent description of their methods in their publication of their original results. We should not coddle authors of scientific papers: they should expect attempted replications as inevitable, contingent upon how much effort replication would take and the credibility being attached to their findings.

### Pre-approval by peer review of attempted replications

The strong recommendation is that investigators planning to attempt a replication should first get pre-approval by independent peer review – including the authors of the original research – of their rationale, design, and analytic plans. Again, why adopt such cumbersome rules if publication of the original research was not subject to them? Peer review can be a slow, undependable process that may introduce biases, not only from the original investigator but of theoretical and professional allies. John Ioannidis’ concept [36] of *obligated replication* comes to mind. This refers to a corruption of peer review whereby proponents of a dominant school of thought or theory control publication venues so they can largely select and mold what gets published. This requirement of prior peer review of replication initiatives inadvertently extends their control to even what research can be re-evaluated.

### Direct, rather than conceptual replication

Whether direct replication is preferred to conceptual replication or whether internal versus external validity is to be emphasized depends a lot on context. It is common practice going back to Berkowitz and Donnerstein [37] for social psychologists to insist on the tightest of experimental procedures while at the same time claiming broadest generalizability to the same world. Fraudster Diederik Stapel [38] claimed that before he resorted to outright fabrication of data, he wrote to investigators when he could not replicate their striking findings. He often got advice, such as:

“Don’t do this test on a computer. We tried that and it doesn't work. It only works if you use pencil-and-paper forms.”

“This experiment only works if you use ‘friendly’ or ‘nice’. It doesn't work with ‘cool’ or ‘pleasant’ or ‘fine’. I don’t know why.”

Amazed that he could now replicate the results, Stapel considered himself as admitted to the “Grand Fellowship of Secret Procedures.”

Any investigator who has been in the field for very long has realized that minor, seemingly arbitrary and even theoretically irrelevant modifications in procedures can lead to a considerable difference in the size and direction of results that are obtained. Insistence on direct replication as a general principle rather than a strategy requiring justification could perpetuate acceptance of results of only limited generalizability. The issue becomes more important when social or public health implications are claimed for findings.

For instance, a bug killing paradigm [39] has been used to make socially important generalizations about soldiers being put at risk for posttraumatic stress disorder when they are placed in morally injurious situations. Arguably, investigators attempting replications should not be confined to the specific species of insects as the original experiments, given the robustness and broad generalizations claimed for the original study. If the replicators fail with different insects, post-publication peer reviewers are free to dismiss any utility of pursuing this line of research – or to applaud it. Similar situations are posed by researchers who claim in heavily promoted studies that positive thinking saps energy and initiative in everyday life, based on studies of undergraduate females having their satisfaction with the hypothetical purchase of high heels assessed in interaction with a computer [40]. Given the common undeclared conflicts of interest of these investigators and such a broad claim of generalizability claimed for everyday life, skepticism should be encouraged and not constrained by these researchers’ subsequent claims of lack of fidelity to the often fragile or poorly defined original experimental conditions.

Reversing the traditional perspective that a psychology study should be tightly controlled in artificial laboratory situations, replicators might consider deliberately loosening experimental control with the intention of incorporating more real-world elements and testing the generalizability of claims across variations. Experimental realism and simulations of the context which generalizations are made should trump original investigators’ opinions about the fidelity of replications to the original manipulations.

### Protecting premium top shelf journals from null findings and attempted replication

The Open Science Collaboration’s attempted replication of 100 studies was published in the prestigious journal, *Science*. Publishing the first paper from the replication initiative was consistent with the journal’s policy of valuing the newsworthy and innovative. Yet, we should be skeptical about whether publishing a bundled set of 100 attempted replications of studies in prestigious psychology journals is a game-changing precedent that will result in routine publication of smaller collections or a single replications in premium top shelf journals. *Science* is a prime example of a journal that has earned its “premium top shelf” status by not routinely publishing replications or null findings unless there is some extraordinary reason for doing so.

The prestigious psychology journals that published the original studies slated for The Open Science Collaboration effort – *Journal of Personality and Social Psychology, Psychological Science,* and *Journal of Experimental Psychology: Learning, Memory, and Cognition*- are unlikely anytime soon to give routinely attempted replications, particularly those producing null results, the same priority as original research -which the RP:P suggested is untrustworthy. An outgoing editor of *Psychological Science* [41] stated that he had rejected over 6000 submissions in his five years as editor without the manuscript is going out to reviewers. At the top of his three reasons was:

*“The Pink Floyd Rejection:* Most triaged papers were of this type; they reported work that was well done and useful, but not sufficiently groundbreaking. So the findings represented just *another brick in the wall* of science.”

Praise of “Pink Floyd rejection” can be turned into a critique of a particular type of publication bias that characterizes *Journal of Personality and Social Psychology* as well as *Psychological Science.* It can serve as a warning that replications of individual published studies, particularly those that do not yield positive results, are not welcomed. But such “bricks in the wall” are likely more trustworthy than the over 50 % of *Journal of Personality and Social Psychology* and *Psychological Science* articles evaluated in the RP:P that did not reproduce with the same strength of effects.

A number of compromises have been struck between organized efforts to further replicate studies in the psychological literature and professional organization publishers. Both the American Psychological Association and the Association for Psychological Science have endorsed replication initiatives, but direct them to journals other than their protected premium top shelf journals.

These compromises serve to protect the strong publication bias and therefore the unrepresentativeness of what is published in these premium top shelf journals. The prestige of *JPSP* and *PS,* as reflected in the journal impact factors by which these two journals compete against each other, is furthered by keeping out individual replications, especially those with null findings. The validity of journal impact factors has of course been subject to withering criticism, but they still matter to early career investigators attempting to advance. Deals between replication initiatives and the APS protect *Psychological Science* from having to accept individual replications, positive and failed, by requiring preregistration and gathering replications up and herding them into a ‘ghetto’ in *Perspectives on Psychological Science*. For the APA, *Journal of Consulting and Clinical Psychology* gets similar protection by exiling null psychotherapy trials in a special section of brief reports in the less prestigious and lower impact *Journal of Psychotherapy Integration.* Successful and failed replications of studies originally published in the APA’s *Journal of Personality and Social Psychology* are referred on to the non-APA journal *Social Psychology*. The energy of researchers seeking to improve the trustworthy of psychologists are deflected from continued demands for enforcement of the Pottery Barn rule (https://hardsci.wordpress.com/2012/09/27/a-pottery-barn-rule-for-scientific-journals/): journals which publish original research should be required to publish attempted replications.

### The right target: questionable publishing practices rather than questionable research practices

Replication initiatives implicitly place the proximal cause of untrustworthiness of psychological science in endemic questionable research practices. Various list and taxonomies of QRP’s are available, but Simmons, Nelson, and Simonsohn’s [42] list of six ways to p-hack are a useful start, even if incomplete:

1. Stop collecting data once *p* < .05.
2. Analyze many measures, but report only those with *p* < .05.
3. Collect and analyze many conditions, but only report those with *p* < .05.
4. Use covariates to get *p* < .05.
5. Exclude participants to get *p* < .05.
6. Transform the data to get *p* < .05.

Although there is a general squeamishness about blaming authors of individual papers, replication initiatives are needed because of the high prevalence of these QRPs in the psychological literature, even in the prestigious journals which the RP:P sampled. Replication initiatives essentially expose the QRPs in published research by demonstrating that key findings cannot be reproduced when independent investigators commit themselves to transparently plan, conduct, and report their replication efforts.

But authors have incentives and protections for engaging in QRPs from strong institutional pressures to publish noteworthy, immediately newsworthy, and ostensibly novel findings versus findings that are more robust but more mundane. As long as pressure on authors from institutions continues, replication initiatives waste the effort of investigators in who might otherwise commit themselves to moving science ahead by building on the secure foundation of more trustworthy past research.

Much could be accomplished by insisting on diligent enforcement of existing rules and standards of best publication practices. Psychology has tended to take its cue from reforms in the biomedical literature where compliance, even though far from perfect, is more likely because of the pressures of government and regulatory agencies that insist on compliance as a condition for approval of pharmaceuticals and medical devices. Psychological journals adopted Consolidated Standards of Reporting of Trials (CONSORT) [43] later and less consistently than medical journals did. Until my colleagues and I protested [44], the American Psychological Association’s late adoption of CONSORT applied only to randomized evaluation of psychological interventions that were explicitly labeled randomized trials in the title or abstract. But that a randomized trial is labeled as such is a checklist item of the CONSORT checklist, not a condition under which the checklist is applicable.

Requirements that the rationale, design, analytic plans, and primary outcomes of clinical trials be registered are similarly being only slowly and inconsistently adopted for psychological interventions. There is evidence that trial registration, if it takes place at all, is after data collection has begun [45]. There is further evidence that editors and reviewers fail to consult published trial registration and protocols in evaluating manuscripts, with the effect that primary outcomes often shift in the published reports [46]. Requests for sharing of data when sharing is mandated are often rejected or simply ignored, with evidence that authors of studies with exaggerated interpretation of findings or outright errors are less responsive to requests for their data [47].

## Lessons learned from challenging untrustworthy psychology

I offer the following three instances of my colleagues and I probing the untrustworthiness of the psychological literature not as anecdata, but as prompts for anyone to reproduce our efforts with other papers to see if evidence of similar questionable publication practices can be elicited. In each instance, we got locked into a time-consuming process with unsatisfactory outcomes and the initial claims continued to be cited. Yet, in each instance, we also demonstrated that any effort at replication would have been wasteful. It would also have been even more time-consuming. The simple lesson to be learned is that we should not depend on replication initiatives when there is a more pressing need to implement and insist upon basic reform of publication practices. But we also learned of the barriers to correction of questionable science, and the ineffectiveness of letters to the editor as a means of post publication peer review. In each instance, submitting a letter to the editor marked the beginning of an unsatisfactory long-term relationship with the authors being criticized.

### Example 1

My colleagues and I were skeptical of effects claim for a loving-kindness meditation intervention on physical health [48] that was heavily publicized in the popular press and cited as the science behind one of the senior author’s workshops. There was no mention in the article that the study was a randomized trial. This was only revealed, as a purported strength of the study, in a popular book written by the senior author. The report lacked basic features required by a CONSORT checklist assessment of a manuscript being submitted for publication. Reanalysis of the data revealed that apparent evidence of an effect in complex secondary analyses was due to an inexplicable deterioration in the control group. We wrote a letter to the editor [49] which was initially rejected with the associate editor stating she would not allow us to engage in a “witch hunt.” Intending to appeal this decision, we discovered that contrary to the requirements of Committee on Publication Ethics (COPE), the journal did not specify a formal appeal process. Our letter was eventually accepted, but the authors got the last word in a letter [50] that did not address the issues we raised. So, we uncovered (1) the journal did not endorse CONSORT nor adhere to COPE guidelines; (2) a high threshold had been set for any correction through the post-publication peer review of letters to the editor; and (3) the authors did not have to meet the same standards in replying. Finally, when the editor was alerted to the financial conflict of interests of the authors, no correction or erratum was issued. The first author of the target article has left the original laboratory and although continuing this line of research, no longer cites the target article.

### Example 2

I wrote a letter to the editor [51] concerning a paper in a prominent journal that reported extraordinary effects of psychological processes on physical health through modification of gene expression [52]. The paper had been highly publicized in the popular media and was cited in workshops and webinars as the key evidence for adopting particular life strategies. The authors responded with a dismissive and evasive reply [53]. My colleagues and I then obtained their data and demonstrated the same results could be obtained by entering random numbers into the equations. We also found some seemingly minor errors in data coding and entry that turned out to have profound effects in our reanalysis. The authors wrote another dismissive response [54] and then altered what had been the publicly available data set and re-analyzed their data and published it elsewhere [55] with a harshly critical attack on us. However, using date-stamped downloads of their data, we were able to determine that the critiques of us depended on integrating the altered dataset with analyses based on the unaltered, original data set. Both journals have refused to retract these papers.

### Example 3

There has been international criticism of the PACE clinical trial examining cognitive behavioral therapy for chronic fatigue syndrome [56]. The authors switched outcomes after the start of the trial. The disputed interpretation of their results favored their declared interests. A colleague and I wrote a letter to the editor [57] and we had the usual unsatisfactory reply from the authors [58].

Critics had made numerous requests to examine the data, consistent with national and international recommendations for data sharing. However, all requests have thus far met with refusals under the UK Freedom of Information Act (FOIA). I attempted to circumvent previous refusals to share data by making a formal request for data from a related paper [59]. The difference was the data from this paper were promised to be available as a condition for publishing in *PLOS One*. The authors responded by turning my journal-level request into a Freedom of Information Act (FOIA) request – which it was not – as well as attacking my character and motives for requesting the data. I turned the matter back to *PLOS One*, and after many months the issue is still not resolved. This remains an ongoing saga. Months after my initial request, I have not obtained the data.

## Developing social media as an alternative to overreliance on replication initiatives

It is impossible to quantify the prevalence of questionable research practices and questionable publication practices from results of replication initiatives. We cannot estimate the needed, but unknown denominator of what is left unpublished, distorted beyond recognition in the prepublication peer review process, or left unwritten because results cannot be suitably spun to fit a confirmation bias. We cannot even estimate the incidence of bruising encounters with the publication process like the three I just described. In most cases, no trace appears in the literature, particularly when initial letters to the editor are rejected, often because of editorial practices allowing authors veto power over publication of criticism.

I have generated attention on social media to my frustrated efforts to obtain data sets that should have been available because of the conditions under which funding was obtained for papers which were published. One basic function of social media is whistleblowing, calling attention to such lapses in best practices, begin classifying and quantifying them, and to mobilize efforts at reform.

However, as seen in important developments like PubPeer and PubMed Commons, social media can provide effective and timely post publication peer review. Attention on social media can either prompt authors and journals’ self correction or provide a correction of the subsequent record when, not infrequently, self correction is refused. In contrast to the drawn out unrewarding processes into which my colleagues and I fell, there are recent self-organizing threads of criticism on PubPeer that have prompted notable postings of corrections and even retraction within weeks or months. It would be an interesting exercise for someone to examine the 100 papers which the RP:P attempted to replicate and come to some consensus as to whether attempted replication would be worth the effort. I strongly suspect for many of these papers, it would not be. So, future replication initiatives – and they will inevitably occur – should adopt such crowd sourced evaluation and preselection to avoid wasting anonymous resources on work that is simply not worth replicating.

Both PubPeer and PubMed Commons allow response from authors whose work is criticized, but neither allows these authors control ceded by the RP:P or conventional letters to the editor. Both are unfettered in just the way that is needed at this point of crisis in the trustworthiness of psychology. PubPeer allows an anonymity that outrages some authors and offends some critics. Yet, a strong argument can be made that retaliation against junior academics voicing criticism on Twitter warrant anonymity, but regardless, this important feature shows no signs of going away in the near future.

PubMed Commons requires commentators to register and identify themselves and so is an important complement to PubPeer. PubMed Commons will eventually be providing appropriate citation, access by search engines, and credit in ways that can contribute to junior academics' career development. Overcoming the untrustworthiness of psychology will be a long, uncertain, and often thankless task, meeting with predictable but also unanticipated resistance. But reform can be accelerated by the strategic use of social media and may, in fact, depend on it.
